# Dynapenia and Sarcopenia in Post-COVID-19 Syndrome Hospitalized Patients Are Associated with Severe Reduction in Pulmonary Function

**DOI:** 10.3390/jcm12206466

**Published:** 2023-10-11

**Authors:** Arturo Orea-Tejeda, Robinson Robles-Hernández, Dulce González-Islas, Luz Jimenez-Gallardo, Laura Gochicoa-Rangel, Armando Castorena-Maldonado, Rafael Hernández-Zenteno, Alvaro Montañez-Orozco, Benigno Valderrábano-Salas

**Affiliations:** 1 Heart Failure and Respiratory Distress Clinic, Cardiology Service, Instituto Nacional de Enfermedades Respiratorias “Ismael Cosío Villegas”, Mexico City 14080, Mexico; oreatart@gmail.com (A.O.-T.);; 2 Department of Research in Tobacco Smoking and COPD at Instituto Nacional de Enfermedades Respiratorias “Ismael Cosío Villegas” 2, Mexico City 14080, Mexico; robinsonrobher@gmail.com; 3 Department of Pulmonary Physiology at Instituto Nacional de Enfermedades Respiratorias “Ismael Cosío Villegas”, Mexico City 14080, Mexico; 4 Direction for Medical Care in Pneumology at Instituto Nacional de Enfermedades Respiratorias “Ismael Cosío Villegas” 4, Mexico City 14080, Mexico; 5 COPD Clinic at Instituto Nacional de Enfermedades Respiratorias “Ismael Cosío Villegas”, Mexico City 14080, Mexico

**Keywords:** post-COVID-19 syndrome, dynapenia, sarcopenia, obesity, pulmonary function, respiratory muscle strength

## Abstract

Background: After hospital discharge, post-COVID-19 syndrome has been observed to be associated with impaired diffusing capacity, respiratory muscle strength, and lung imaging abnormalities, in addition to loss of muscle mass/strength, sarcopenia, and obesity impact exercise tolerance, pulmonary functions, and overall prognosis. However, the relationship between lung function and the coexistence of obesity with low muscle strength and sarcopenia in post-COVID-19 patients remains poorly investigated. Therefore, our aim was to evaluate the association between lung function and the coexistence of obesity with dynapenia and sarcopenia in post-COVID-19 syndrome patients. Methods: This cross-sectional study included subjects who were hospitalized due to moderate to severe COVID-19, as confirmed by PCR testing. Subjects who could not be contacted, declined to participate, or died before the follow-up visit were excluded. Results: A total of 711 subjects were evaluated; the mean age was 53.64 ± 13.57 years, 12.4% had normal weight, 12.6% were dynapenic without obesity, 8.3% had sarcopenia, 41.6% had obesity, 21.2% had dynapenic obesity, and 3.8% had sarcopenic obesity. In terms of pulmonary function, the dynapenic subjects showed decreases of −3.45% in FEV_1_, −12.61 cmH2O in MIP, and -12.85 cmH2O in MEP. On the other hand, the sarcopenic subjects showed decreases of −6.14 cmH2O in MIP and −11.64 cmH2O in MEP. The dynapenic obesity group displayed a reduction of −12.13% in PEF. Conclusions: In post-COVID-19 syndrome, dynapenia and sarcopenia—both with and without obesity—have been associated with lower lung function.

## 1. Introduction

Post-COVID-19 syndrome is defined as the presence of symptoms and abnormalities persisting beyond twelve weeks from the onset of the acute viral infection caused by SARS-CoV-2, with no alternative diagnoses [1]. Declined diffusing capacity of the lung for carbon monoxide (DLCO), respiratory muscle strength, and lung imaging abnormalities have been observed in COVID-19 patients during the early convalescence phase following hospital discharge [2]. Additionally, patients experiencing persistent dyspnea exhibit significant restrictions on spirometry and lower DLCO and functional capacity [3].

In the general population, pulmonary function, specifically the reduction in forced expiratory volume in 1 s (FEV_1_), predicts mortality [4]. Various factors have the ability to influence pulmonary function, including sex; age; immune system alterations; exposure history to toxic agents, such as tobacco smoke, wood smoke, and asbestos; and muscular strength [5,6]. The pathophysiology process involves oxidative stress, hypoxia, inactivity, malnutrition, a higher catabolic condition, and glucocorticoid use [7].

Physical fitness depends on muscular strength and plays an independent role in preventing and treating chronic diseases, particularly in older individuals [8]. Low muscle mass significantly impacts human life, leading to several conditions, including more postoperative complications, prolonged hospital stays, and worse physical function and quality of life [9].

Two meta-analyses have demonstrated that muscular strength predicts mortality and cardiovascular diseases [9,10,11]. In addition, in post-COVID-19 patients in the intensive care unit, impairment in cardiovascular function is common, especially right ventricle dysfunction, and is associated with a worse prognosis [12,13]. In addition, subjects with lower muscular strength have a 1.8-fold higher risk of death than those with conserved muscular strength [14]. During aging, a generalized loss of muscle mass and strength is closely linked to mitochondrial muscle dysfunction; this loss includes respiratory muscles, such as the diaphragm and intercostal muscles, with reduced lung function [6,15].

In cardiovascular and respiratory systems, the functional capacities decrease with a decrease in mitochondrial volume. This decrease is often a result of age and several chronic diseases [16]. It is essential to remark that body mass index (BMI) and cardiorespiratory fitness play an important role in developing muscle mitochondrial dysfunction due to aging [17]. Consequently, muscular strength can serve as a prediction marker for adverse events and enable early intervention strategies for patients with muscle anabolism [18]. Muscle wasting is associated with decreased pulmonary function [19,20]. In addition to this, the strength of the intercostal muscles and the diaphragm affects pulmonary function [21]. In chronic obstructive pulmonary disease (COPD), lower skeletal muscle mass is related to less functional capacity and muscle strength, pulmonary abnormalities, and a poor prognosis [22,23].

Sarcopenia is a widespread and progressive skeletal muscle disorder that manifests as reduced mass and functional muscle, increasing the risk of several adverse events [24]. Sarcopenia can be classified as “primary”, or age-related, and “secondary” when a systemic disease, especially one that may cause inflammatory processes, is a causative factor.

The loss of muscle mass/strength, sarcopenia, and obesity have multifactorial origins that are strongly interconnected and mutually exacerbate each other. This creates a vicious cycle, which includes several factors, such as mitochondrial abnormalities, declined protein synthesis, inadequate essential amino acid intake for protein synthesis, hypoxemia that inhibits protein synthesis, and raised proteolysis due to a pro-inflammatory state [24].

On the other hand, adipose tissue plays a central role in pulmonary function. Obesity, especially central obesity, is independently associated with worse pulmonary function; this decline in respiratory function and central obesity may be associated with restrictive patterns of obstructive alterations [25,26,27].

However, the relationship between lung function and the coexistence of obesity with low muscle strength and sarcopenia in post-COVID-19 subjects is not well established. Our aim is to evaluate the association between lung function and the coexistence of obesity with dynapenia and sarcopenia in post-COVID-19 syndrome patients.

## 2. Materials and Methods

A cross-sectional study was conducted at the Instituto Nacional de Enfermedades Respiratorias “Ismael Cosío Villegas” in Mexico City. The data were obtained from out-patient evaluations conducted three months after individuals had experienced acute COVID-19 infection. These evaluations were part of routine clinical examinations for post-COVID-19 subjects between 1 June 2020 and 30 May 2023.

The study focused on moderate to severe COVID-19 patients who had a confirmed diagnosis through PCR testing. Moderate to severe COVID-19 patients were considered to be those patients who required hospitalization with a blood oxygen saturation level of 92% or lower and a PaO2/FiO2 ratio less than 150 (which represents the arterial partial pressure of oxygen divided by the fraction of inspired oxygen). Further, the patients included in the study had required hospitalization and were subsequently discharged. Subjects who could not be contacted, declined to participate, or died before the follow-up visit were excluded.

The study was conducted in accordance with the Declaration of Helsinki and approved by the Ethics Committee of the Instituto Nacional de Enfermedades Respiratorias “Ismael Cosío Villegas” (approval number: C71-20).

### 2.1. Outcome Measures

As part of our comprehensive post-COVID-19 clinical management, we conducted evaluations on clinical and demographic variables for the patients who visited our institute. In addition, to evaluate the relationship between lung function, dynapenia, sarcopenia, and obesity in individuals with post-COVID-19 syndrome, we assessed the following outcome measures:

### 2.2. Anthropometry 

Weight and height were assessed following the manual anthropometric standardization reference [28]. All subjects were dressed in light clothing and were barefoot. BMI was estimated by dividing each individual’s total body weight (in kilograms) by the square of their height (in meters).

### 2.3. Handgrip Strength

Handgrip strength was measured using a mechanical Smedley Hand Dynamometer (Stoeing, Wood Dale, UK) in compliance with the technique described in Rodriguez et al. [29].

### 2.4. Body Composition

Body composition was estimated with whole-body bioelectrical impedance utilizing four-pole mono-frequency equipment, namely, the RJL Quantum IV analyzer (RJL Systems^®^, Clinton Township, MI, USA). The standard technique [30] was employed, ensuring consistency and accuracy. All measures were performed by the same operator in the morning within a comfortable environment devoid of drafts and equipped with portable electric heaters. To maintain the integrity of the results, the patients were recommended to fast and abstain from exercise for eight hours prior to the study. They were also requested to abstain from consuming alcohol 12 h before the study. Throughout the duration of the study, the subjects remained in a supine position, with their arms positioned at about 30° from the trunk and their legs separated at about 45°. The designated area was thoroughly sanitized with alcohol before electrodes were placed on the hand and ipsilateral foot. Fat mass was estimated by the RJL Systems BC 4.2.2 software.

Additionally, appendicular skeletal muscle mass (ASM) was evaluated using Sergi’s equation [31]: ASM (kg/m^2^) = [−3.964 + (0.227 × (Height2 (cm)/Resistance) + (0.095 × Weight) + (1.384 × Sex) + (0.064 × Reactance)/Height (m^2^)].

### 2.5. Body Composition Classification 

The criteria for determining normal weight were defined as adequate muscle mass (ASM > 7.0 kg/m^2^ in men and ASM > 6.0 kg/m^2^ in women), muscle strength (handgrip strength > 27 kg in men and handgrip strength > 16 kg in women), and fat mass (<25% in men and <35% in woman).

Dynapenia without obesity was characterized by low muscle strength, as defined by the European Working Group on Sarcopenia in Older People (EWGSOP2) [24]. Specifically, men were considered to have dynapenia if their handgrip strength was below 27 kg and women if their handgrip strength was below 16 kg. Additionally, fat mass had to be below 25% for men and below 35% for women.

Sarcopenia without obesity was defined according to the EWGSOP2 guidelines as low muscle mass (ASM < 7.0 kg/m^2^ in men and ASM < 6.0 kg/m^2^ in women), low muscle strength (handgrip strength < 27 kg in men and handgrip strength < 16 kg in women), and fat mass (<25% in men and <35% in women) [24,32].

Obesity was defined as fat mass greater than 25% in men and greater than 35% in women [29].

Dynapenic obesity was diagnosed in men with a handgrip strength below 27 kg and fat mass greater than 25%, and in women with a handgrip strength less than 16% and fat mass above 35% [24,32].

Sarcopenic obesity was defined as low muscle mass (ASM < 7.0 kg/m^2^ in men and ASM < 6.0 kg/m^2^ in women), low muscle strength (handgrip strength < 27 kg in men and handgrip strength < 16 kg in women), and excess adiposity (fat mass > 25% in men and fat mass > 35% in women) [24,32].

### 2.6. Pulmonary Function 

Spirometry testing was performed by an experienced respiratory medicine technician operating a portable spirometer (EasyOnePC, Ndd Medical Technologies Inc., Zürich, Switzerland) in compliance with the standards of the American Thoracic Society/European Respiratory Society (ATS/ERS) [33]. The spirometry variables analyzed included the forced expiratory volume in the first second (FEV_1_) and the forced vital capacity (FVC), maximal mid-expiratory flow rate (MEF 25–75), and peak expiratory flow (PEF) before and after administration of a bronchodilator. Following a 15 min rest period, each participant was instructed to perform a maximum forced inhalation and a powerful forced expiration while wearing a nose clip. The reference values for spirometry were obtained specifically for Mexican-American individuals [34]. 

Respiratory muscle weakness was defined as PEF < 4.40 L/s for men and PEF < 3.21 L/s for women [35].

### 2.7. Respiratory Muscle Strength 

The maximum inspiratory pressure (MIP) and maximum expiratory pressure (MEP) were measured based on the ATS/ERS 2002 guidance using the MicroRPM equipment (CareFusion, Micromedical, UK) [36].

### 2.8. Statistical Analysis 

The data analyses were performed using the commercially available package Stata version 14 (Stata Corp., College Station, TX, USA). Qualitative variables were given as frequencies and percentages. To assess the normality of continuous variables, the Shapiro–Wilk test was employed. Normally distributed continuous variables were expressed as mean and standard deviations, while non-normal variables were presented as medians and percentiles 25 to 75. A comparison among the different study groups (normal, dynapenic without obesity, sarcopenia without obesity, obesity, dynapenic obesity, and sarcopenic obesity) was analyzed using X2 for qualitative variables and ANOVA or Kruskal–Wallis tests for continuous variables. To examine the association between pulmonary function and the coexistence of obesity with dynapenia and sarcopenia, a simple linear regression was performed. The multivariate linear regression was adjusted for variables in the bivariate analysis with a significance level of *p* < 0.10: age, sex, obesity, Charlson Comorbidity Index, mechanical ventilation, and hospital stay days. A *p* < 0.05 was assumed to be statistically significant.

## 3. Results

A total of 711 patients were evaluated. The mean age was 53.64 ± 13.57 years. Among the patients, 12.4% had normal weight, 12.6% had dynapenia without obesity, 8.3% had sarcopenia, 41.6% had obesity, 21.2% had dynapenic obesity, and 3.8% had sarcopenic obesity.

The prevalence of dynapenia and sarcopenia was found to be considerably high in our population, especially in those individuals without obesity. However, in both cases, these conditions were more prevalent in older patients compared to those with obesity or normal weight. Regarding comorbidities, the prevalence of diabetes mellitus was also significant, with dynapenia and sarcopenia present in more than one third of both obese and non-obese patients. Further, longer hospitalization periods were common and significantly different between the groups with dynapenia and sarcopenia—with those individuals experiencing the longest hospitalizations. A similar trend was observed with invasive mechanical ventilation, where obese patients had the highest percentage, especially among those with sarcopenia (Table 1).

In terms of pulmonary function, individuals with dynapenia and sarcopenia with and without obesity exhibited lower FEV_1_ (L), FVC (L), DLCO (%), MIP (cmH_2_O), and MEP (cmH_2_O), and PEF (%) than normal-weight subjects. Moreover, subjects with dynapenia with and without obesity, as well as those with sarcopenia, had lower PEF and 6MWT (Table 2).

When performing the lineal regression model, we observed a significant decrease of 3.45% in the predicted FEV_1_, 12.61 cmH_2_O in MIP, and 12.85 cmH_2_O in MEP in subjects with dynapenia. In the case of sarcopenia subjects, the adjusted model revealed decreases in MIP and MEP. However, when considering patients with dynapenia with obesity, we only observed a decrease in PEF. Finally, for sarcopenia–obesity, no significant reductions were found (Table 3).

## 4. Discussion

Pulmonary function can be affected by various factors, such as age, muscular strength, inflammatory processes, and immobility [5,6]. These factors often coexist in COVID-19 patients who have been hospitalized, resulting in severe alterations in muscular tissue that can potentially affect mechanical respiratory function, especially in those individuals with pre-existing comorbidities who require hospitalization, long-stay hospitalization, and invasive mechanical ventilation [37]. The severity of the disease has been associated with an increased frequency of manifestations, such as low muscle mass and pulmonary function [37,38]. In the current study, we evaluated patients three months after acute COVID-19 infection as part of our routine clinical examinations for post-COVID-19 subjects. It is well established that various factors, including acute respiratory distress syndrome severity, age, and comorbidities, can impact mechanical function and diffusion capacity. Our study showed the independent association between dynapenia and FEV_1_, MEP, and MIP after adjusting for confounding variables, such as age, sex, comorbidities, obesity, mechanical ventilation, and hospital stay days, which may be related to the deterioration of physical and nutritional performance commonly observed in critically ill patients during their recovery phases [39]. Although subjects with dynapenia have a lower FEV_1_, this reduction cannot be considered clinically significant, as it is no more than 10% or more than 100 mL.

On the other hand, in our population, dynapenic, dynapenic obesity, and sarcopenic subjects had lower PEF% values than normal weight subjects. In addition, respiratory muscle wasting was 13.2% in sarcopenia, 4.8% in dynapenia, 4.2% in sarcopenic obesity, 3.5% in dynapenic obesity, and 1.5% in obesity subjects. Similar results have been shown in other populations where the respiratory muscle strength was associated with lower muscular strength, muscle mass, and sarcopenia [40,41], and this had a negative impact on the pulmonary function [20,21].

Decreased muscular strength is strongly associated with the loss of skeletal muscle mass, a significant tissue responsible for blood glucose uptake. It functions as an extensive reservoir of amino acids stored as protein. The depletion of protein stores due to this loss is linked to illnesses, such as metabolic dysregulation [42], increased systemic low-grade inflammation [43], and immunosuppression [44].

Maintaining muscle strength throughout life improves the ability to carry out activities of daily living and functional capacity [19]. Handgrip strength has been a good indicator of the overall functionality of muscle [11,45].

Muscle wasting is linked to decreased muscle strength, pulmonary abnormalities, and a poorer prognosis in COPD patients. Diverse studies have indicated that FEV_1_ reduction significantly predicts mortality in the general population [46]. 

The decline in skeletal muscle mass/strength and the evolution of sarcopenia have multiple causes, such as ageing, mitochondrial dysfunction, decreased protein anabolism, diminished essential amino acid intake for protein synthesis, major protein catabolism due to a pro-inflammatory state, and hypoxemia, which interferes with protein synthesis [24].

Acute sarcopenia is typically related to an acute disease. It is characterized by decreased function and muscle mass during hospitalization, whereas chronic sarcopenia is associated with chronic and progressive conditions [14].

In our population, the dynapenia and sarcopenia groups without obesity were the oldest subjects. The loss of muscle mass and strength occurs with the aging process. It is estimated that in subjects aged 18–45 years, the loss of muscle mass is 0.47% per year in men and 0.37% per year in women, while in subjects older than 75 years, it is 0.64 to 0.70% per year in women and 0.80 to 0.98% per year in men [47].

In addition, a decrease in hand strength is associated with reduced respiratory muscle strength in both MIP and MEP, the former being an indicator of diaphragm strength. At the same time, the latter assesses the strength of the abdominal and intercostal muscles [48].

This decline in respiratory function due to aging was defined by Nagano et al. as presbypnea [49]. In addition, subjects with sarcopenia had the highest respiratory muscle weakness (Table 2).

In our population, sarcopenia was negatively associated with low FEV_1_, MIP, and MEP after adjusting for confounding variables. Similar results have been observed in other populations. In patients with pulmonary disease like COPD patients, for every 1 kg/m^2^ of ASM, there was an increase of 4.89% in FEV_1_. And for every 1 kg/m^2^ of ASM, there was an increase of 2.87% in FEV_1_ [50]. These results were strengthened by Park et al., who discovered that muscle wasting was associated with a risk of FEV_1_ lower than 80% (OR: 2.97, CI 95% 2.74 to 3.17) and FVC less than 80% in subjects without pulmonary disease [20]. Although these results cannot be extrapolated to post-COVID-19 patients, they show the impact of muscle mass on lung function.

On the other hand, the association between dynapenia or sarcopenia and low DLCO is not clear; some studies have shown that it may be the effect of hypoxia and the severity of the lung disease that leads to dynapenia or sarcopenia, since it has been found in patients with other chronic lung diseases [51]; but, in our study, its relationship with patients in a post-critical state does not entail any association.

In the present study, both dynapenia and sarcopenia were present and linked to poor pulmonary function. Additionally, these conditions had a significant effect on the duration of hospitalization. Moreover, we found statistically significant differences in pulmonary function between patients with dynapenia and sarcopenia. Both dynapenia and sarcopenia negatively impacted the course of hospitalization and mechanical ventilation and decreased FEV_1_ [52,53]. This allows us to deduce that the decrease in MIP and MEP is due to a decline in respiratory muscular strength from the loss of muscle mass [48]. 

Some studies have shown that obesity, determined by BMI, positively affects lung function [54] and prognosis in diverse populations [55,56], which has been termed the “obesity paradox”. However, it is essential to mention that BMI cannot accurately distinguish between the two most significant components of body composition: fat mass and fat-free mass, specifically ASM, which is essential for lung function. Our study evaluates the impact of dynapenia, sarcopenia, and obesity assessed by body composition methods on lung function. 

Our study shows that subjects with dynapenia and sarcopenia have lower lung function compared to subjects with normal weight and obesity, and these populations are different in terms of age and clinical variables; this is evidence that clinical practice must consider these body composition phenotypes as independent populations. For this reason, our study performed a multivariate analysis adjusted for confounding variables, showing that dynapenia and sarcopenia, independently of obesity, negatively impacted respiratory muscle strength assessed by MIP and MEP. Furthermore, the interaction between dynapenia and obesity was observed to reduce PEF%, which could suggest adipose tissue infiltration into muscle, which promotes myofibril atrophy and loss of muscle function [57,58].

Our research outcomes suggest that those with less muscle mass and strength actually exhibit poorer respiratory function. In addition, there is a 22% prevalence of malnutrition in post-COVID-19 patients, which increases in those subjects who require hospitalization and are over 65 years [59]. Malnutrition is due to anorexia, inflammation, prolonged hospital stays, and mechanical ventilation requirements in some subjects [60]. 

This highlights the urgent need for therapeutic measures to mitigate and reverse the loss of muscle mass and strength, thus preventing deterioration of lung function. 

Nutritional treatment and pulmonary rehabilitation are the cornerstones of managing dynapenia and sarcopenia. Regarding nutritional treatment, supplementation with L-arginine plus vitamin C in adults with post-COVID-19 improved walking performance, muscle strength, endothelial function, and fatigue in adults with post-COVID-19 [61]. Similarly, supplementation with beta-hydroxy-beta-methylbutyrate, a derivative of leucine, a branched-chain amino acid, has increased strength and skeletal muscle mass [62,63]. On the other hand, monounsaturated fatty acids, mainly oleic acid fatty, have a positive impact on inflammatory factors, endothelial function, as well as oxidative stress [64,65]. With respect to pulmonary rehabilitation, in healthy older adults, inspiratory muscle training has been shown to improve inspiratory muscle strength [66]. Similarly, respiratory muscle and lung function improvements have been observed in subjects with COPD [67]. In patients with post-COVID-19 syndrome, an improvement in physical function and quality of life and reduction in dyspnea has been observed. 

### Strengths and Limitations of the Study

This study possesses inherent limitations due to its cross-sectional design, such as not being able to determine causality between variables, and the small sample size could impact the representativeness of the data. Due to the nature of our study, we did not evaluate the impact of dynapenia, sarcopenia, and their interaction with obesity and their impact on prognosis. The criteria for diagnosing sarcopenia have not been universally established; however, different consensuses indicate an algorithm based on reduced muscle strength or function and a reduction in muscle mass [24].

Muscle strength can be measured by handgrip strength, and for muscle function, gait speed and chair standing tests have been suggested. Body composition assessment techniques, such as electrical bioimpedance or dual X-ray absorptiometry, can be used to estimate muscle mass. 

An important limitation of this study is that the diagnosis of sarcopenia was made according to the EWGSOP2 consensus [24], which is focused on older persons, and our study included subjects younger than 60 years. This could have led to misclassification bias. The EWGSOP2 guidelines recommend using a cut-off point of two standard deviations below the mean of the healthy adult reference population [24]. Nevertheless, these reference values were unavailable in our population. 

However, one notable strength of this study is that it is the first to investigate the association between body composition, specifically the loss of muscle mass and respiratory function, in post-COVID-19 hospitalized patients.

## 5. Conclusions

In post-COVID-19 syndrome, dynapenia and sarcopenia—both with and without obesity—have been associated with lower lung function. This distinction could be useful for respiratory evaluation of patients with dynapenia, but more studies are required.

## Figures and Tables

**Table 1:** Demographic and clinical characteristics in post-COVID-19 patients.

	Normal Weight *n* = 88, 12.4%	Dynapenia without Obesity *n* = 90, 12.6%	Sarcopenia without Obesity *n* = 59, 8.3%	Obesity *n* = 296, 41.6%	Dynapenic Obesity *n* = 151, 21.2%	Sarcopenic Obesity *n* = 27, 3.8%	*p*-Value between Groups
Age, years	53.5 ± 12.8	61.2 ± 11.2 †‡	64.1 ± 13.4 †‡¶	48.2 ± 11.30 †	54.6 ± 12.4 ‡§	59.6 ± 14.4 ‡	<0.001
Male, *n* (%)	65 (73.8)	70 (77.7)	30 (50.8)	184 (62.1)	88 (58.2)	18 (66.6)	0.003
Comorbidities							
Diabetes, *n* (%)	24 (27.2)	46 (51.1)	25 (42.3)	82 (27.7)	57 (37.7)	8 (29.6)	<0.001
Hypertension, *n* (%)	24 (27.2)	39 (43.3)	26 (44.1)	99 (33.4)	69 (45.7)	10 (37.0)	0.026
Obesity, *n* (%)	0 (0)	0 (0)	0 (0)	216 (72.9)	110 (72.8)	1 (3.7)	<0.001
Ischemic cardiopathy, *n* (%)	5 (5.6)	9 (10)	5 (8.4)	16 (5.4)	14 (9.2)	2 (7.4)	0.562
Pulmonary disease, *n* (%)	11 (12.5)	16 (17.7)	10 (16.9)	41 (13.8)	30 (19.8)	3 (11.1)	0.516
Thyroid disease, *n* (%)	6 (6.8)	4 (4.4)	4 (6.7)	14 (4.7)	12 (7.9)	2 (7.4)	0.771
Hepatopathy, *n* (%)	2 (2.2)	1 (1.1)	0 (0)	6 (2.0)	8 (5.3)	1 (3.7)	0.190
HIV, *n* (%)	0 (0)	2 (2.2)	2 (3.4)	3 (1.0)	2 (1.3)	0 (0)	0.491
Asthma, *n* (%)	2 (2.2)	1 (1.1)	0 (0)	13 (4.4)	6 (3.9)	2 (7.4)	0.282
COPD, *n* (%)	0 (0)	1 (1.1)	3 (5.1)	4 (1.3)	1 (0.6)	2 (7.4)	0.021
CCI > 4, *n* (%)	4 (4.5)	21 (23.3)	17 (28.8)	14 (4.7)	19 (12.6)	8 (29.6)	<0.001
Hospital parameters							
Length of hospital stay, d, median [IQR]	12.5 (0.5–21)	19 (10–33) †	27 (15–48) †‡	14 (8–23)	23 (13–35) †‡	26 (12–46) †‡	<0.001
VMI, *n* (%)	38 (44.2)	62 (68.9)	41 (69.5)	157 (53.2)	116 (77.3)	22 (81.5)	<0.001

HIV, human immunodeficiency virus; COPD, chronic obstructive pulmonary disease; CCI, Charlson Comorbidity Index; VMI ventilation mechanical invasive. Post hoc test: † *p* < 0.05 vs. normal subjects, ‡ *p* < 0.05 vs. obesity subjects, § *p* < 0.05 vs. dynapenic subjects, *p* < 0.05 vs. dynapenic obesity subjects. § *p* < 0.05 vs. pre-sarcopenic subjects, ¶ *p* < 0.05 vs. Pre-sarcopenic obesity subjects, *p* < 0.05 vs. pre-sarcopenic obesity subjects.

**Table 2:** Spirometry parameters according to body composition classification.

	Normal Weight *n* = 88	Dynapenia without Obesity *n* = 90	Sarcopenia without Obesity *n* = 59	Obesity *n* = 296	Dynapenic Obesity *n* = 151	Sarcopenic Obesity *n* = 27	*p*-Value between Groups
Spirometry							
Pre-bronchodilator **							
FEV_1_, L	2.9 ± 0.6	2.4 ± 0.6 †‡	2.2 ± 0.7 †‡	2.9 ± 0.7	2.4 ± 0.7 †‡	2.3 ± 0.7 †‡	<0.001
FEV_1_, %	97.1 (86.2–105)	92.5 (78–103.3)	89.7 (77.5–103.8)	93.2 (84.1–102.2)	88.3 (75.3–102.9) †	89.5 (75.6–102.2)	0.018
FVC, L	3.7 ± 0.8	3 ± 0.7 †‡	2.6 ± 0.8 †‡	3.6 ± 0.8	3 ± 0.8 †‡	2.9 ± 0.9 †‡	<0.001
FVC, %	91.9 ± 14.8	84.8 ± 17.8	83.6 ± 21.1	90.7 ± 17.9	86 ± 18.4	81.5 ± 21.5	0.001
MEF 25–75, %	3.4 ± 1.2	2.9 ± 1.1	3 ± 1.3	3.3 ± 1.1	2.8 ± 1 †‡	3.2 ± 1.2	<0.001
PEF, %	115.4 ± 10.5	101.9 ± 24.2†	97.52 ± 27.6 †‡	109.5 ± 21.0	102.0 ± 24.1 †‡	102.2 ± 23.2	<0.001
FEV_1_/FVC	0.8 ± 0.5	0.8 ± 0.0	0.8 ± 0.0	0.8 ± 0.0	0.8 ± 0.0	0.8 ± 0.0	0.220
Post-bronchodilator **							
FEV_1_, L	3 ± 0.6	2.5 ± 0.5 †‡	2.2 ± 0.7 †‡	2.9 ± 0.7	2.5 ± 0.7 †‡	2.4 ± 0.7 †‡	<0.001
FEV_1_, %	99.9 (88.6–108.4)	92.1 (80.7–103.6)	94.9 (80.2–105.8)	94.7 (85–104)	92.7 (79.7–103) †	92.5 (77.3–103.2)	0.040
FVC, L	3.7 ± 0.8	3 ± 0.7 †‡	2.7 ± 0.9 †‡	3.5 ± 0.8	3.1 ± 0.8 †‡	2.9 ± 0.9 †‡	<0.001
FVC, %	90.8 (83.4–100.4)	86.5 (72.8–96.4) †	84.3 (68.9–95.8)	80.9 (80.3–97.2)	85.9 (74.5–96.6)	86 (69.5–95.2)	0.007
MEF 25–75, %	3.7 ± 1.2	3.1 ± 1.1 ‡	3.3 ± 1.5	3.7 ± 1.3	3.2 ± 1.1 ‡	3.4 ± 1.3	<0.001
PEF, %	117.1 ± 21.9	104.5 ± 25.4 †	93.6 ± 29.1 †‡¶	111.5 ± 21.4	106.6 ± 25.6 †	105.5 ± 27.9	<0.001
FEV_1_/FVC	0.82 [0.7–0.8]	0.82 [0.7–0.9]	0.86 [0.8–0.9] †‡§¶	0.83 [0.8–0.7]	0.83 [0.7–0.8]	0.84 [0.8–0.9]	0.007
Other pulmonary test							
DLCO, % **	78.9 ± 19.1	67.4 ± 20.6 †‡	54.5 ± 22.2 †‡§¶	77.9 ± 21.3	68.9 ± 23.4 †‡	64.5 ± 29.8 ‡	<0.001
MIP, cmH_2_O	106.1 ± 25.5	87.9 ± 22.8 †‡	75.3 ± 26.3 †‡	102.6 ± 25.8	86.6 ± 26.1 †‡	79.2 ± 35 †‡	<0.001
MEP, cmH_2_O	132.1 ± 35.1	107.3 ± 28.8 †‡	87.8 ± 36.4 †‡¶	127.3 ± 36.6	112.7 ± 36.5 †‡	97.6 ± 35.2 †‡	<0.001
6MWT, mts	502.5 ± 85.8	429.4 ± 113.9 †‡	413.9 ± 93.9 †‡	488.6 ± 101.6	407.4 ± 114.4 †‡	460.4 ± 104	<0.001
Respiratory muscle weakness, *n* (%)	0 (0)	4 (4.8)	7 (13.2)	4 (1.5)	5 (3.5)	1 (4.2)	<0.001

FEV_1_, forced expiratory volume in one second; FVC, forced vital capacity; MEF, maximum expiratory flow between 25 and 75%; PEF, peak expiratory flow; DLCO, maximum diffusing capacity of the lung; MIP, maximal inspiratory pressure; MEP, maximal expiratory pressure; 6MWT, Six-Minute Walk Test. Post hoc test: † *p* < 0.05 vs. normal subjects, ‡ *p* < 0.05 vs. obesity subjects, § *p* < 0.05 vs. dynapenic subjects, *p* < 0.05 vs. dynapenic obesity subjects, ¶ *p* < 0.05 vs. Pre-sarcopenic obesity subjects, ** predicted values calculated for Latino population.

**Table 3:** Association between dynapenia, sarcopenia, and obesity with lung function in post-COVID-19 syndrome.

	Crude Model			Adjusted Model		
	β	CI (95%)	*p*-Value	β	CI (95%)	*p*-Value
FEV_1_, % **						
Dynapenia	−3.23	−6.51 to 0.05	0.054	−3.45	−6.89 to −0.02	0.048
Sarcopenia	−0.91	−5.63 to 3.80	0.704	−0.81	−5.55 to 3.92	0.735
Obesity	−1.37	−4.87 to 2.13	0.443	−1.26	−4.83 to 2.30	0.488
FVC, % **						
Dynapenia	−0.52	−0.68 to −0.36	<0.001	−2.63	−6.11 to 0.83	0.137
Sarcopenia	−0.31	−0.54 to −0.08	0.007	−2.66	−7.46 to 2.12	0.275
Obesity	−0.01	−0.18 to 0.15	0.838	−0.49	−4.10 to 3.12	0.790
PEF, % **						
Dynapenia	−2.95	−7.96 to 2.05	0.248	−4.17	−9.37 to 1.03	0.116
Sarcopenia	−1.73	−12.13 to 8.65	0.743	−1.26	−11.57 to 9.04	0.810
Dynapenia + obesity	−12.79	−22.14 to −3.45	0.007	−12.13	−21.59 to −2.68	0.012
Sarcopenia + obesity	−11.58	−25.26 to 2.10	0.097	−9.23	−22.78 to 4.32	0.182
MIP, cmH_2_O						
Dynapenia	−16.02	−20.69 to −11.36	<0.001	−12.61	−17.00 to −8.21	<0.001
Sarcopenia	−11.13	−17.74 to −4.51	<0.001	−6.14	−12.17 to −0.12	0.045
Obesity	−2.65	−7.62 to 2.31	0.295	−2.34	−6.91 to 2.22	0.313
MEP, cmH_2_O						
Dynapenia	−15.66	−22.06 to −9.26	<0.001	−12.85	−18.83 to −6.88	<0.001
Sarcopenia	−19.42	−28.49 to −10.36	<0.001	−11.64	−19.84 to −345	0.005
Obesity	0.85	−5.95 to 7.66	0.805	2.46	−3.74 to 8.67	0.437
DLCO, % **						
Dynapenia	−45.64	−130.74 to 39.45	0.293	−22.11	−114.13 to 69.90	0.637
Sarcopenia	−6.59	−128.62 to 115.43	0.916	8.42	−118.27 to135.12	0.896
Obesity	14.87	−76.33 to 106.08	0.749	17.02	−79.12 to 113.16	0.728

FEV_1_, forced expiratory volume in one second; FVC, forced vital capacity; PEF, peak expiratory flow; PIMAX, maximal inspiratory mouth pressure; PEMAX, peak expiratory pressure; DLCO, maximum diffusing capacity of the lung. Adjusted model by age, sex, obesity, Charlson Comorbidity Index, mechanical ventilation, and hospital stay days. ** Predicted values calculated for Latino population.

## Data Availability

The datasets generated and/or analyzed during the current study are not publicly available due to the fact that individual privacy could be compromised but are available from the corresponding author on reasonable request.
